# Impaired sensitivity to thyroid hormones is associated with frailty in older patients with cardiometabolic disease

**DOI:** 10.1186/s12877-025-06608-y

**Published:** 2025-11-25

**Authors:** Remi Kodera, Yoshiaki Tamura, Yuji Murao, Fumino Yorikawa, Ai Iizuka, Kazuhito Oba, Kenji Toyoshima, Yuko Chiba, Joji Ishikawa, Atsushi Araki

**Affiliations:** 1 Department of Diabetes, Metabolism, and Endocrinology, Tokyo Metropolitan Institute for Geriatrics and Gerontology, 35-2 Sakaecho, Itabashi-ku, Tokyo, 173-0015 Japan; 2 Department of Cardiology, Metropolitan Institute for Geriatrics and Gerontology, 35-2 Sakaecho, Itabashi-ku, Tokyo, 173-0015, Japan; 3 Center for Comprehensive Care and Research for Prefrailty, Tokyo Metropolitan Institute for Geriatrics and Gerontology, 35-2 Sakaecho, Itabashi-ku, Tokyo, 173-0015 Japan

**Keywords:** Thyroid hormone sensitivity, Free thyroxine/Free triiodothyronine ratio, Thyroid feedback quantile-based index, Frailty, Cardiometabolic disease

## Abstract

**Background:**

It is unclear how impaired peripheral and central sensitivity to thyroid hormones relates to frailty. In this cross-sectional study, we investigated whether these indices were associated with the prevalence of frailty in older patients with cardiometabolic disease.

**Methods:**

A total of 637 older patients with cardiometabolic disease who visited an outpatient clinic and were evaluated for thyroid function and frailty indices were included in this analysis. The free triiodothyronine/free thyroxine ratio (fT3/fT4) and Thyroid Feedback Quantile-based Index (TFQI) were measured as peripheral and central sensitivity to thyroid hormones, respectively. Using the Mann–Whitney U test and multivariate logistic regression analysis, we investigated the association between these indices and frailty as defined by the modified Cardiovascular Health Study (mCHS) criteria and the Kihon Checklist (KCL).

**Results:**

The fT3/fT4 was lower and the TFQI was higher in patients with mCHS-defined frailty than in those without frailty (*p* < .001 and *p* = .021, respectively). Patients with KCL-defined frailty had lower fT3/fT4 (*p* < .001) but not TFQI. The fT3/fT4 ratio correlated with age, cognitive function, depressive mood, nutritional status, and physical activity, whereas the TFQI was not. Using multivariate analysis, after adjustment for cognitive function, nutritional status, physical activity, and inflammatory markers, the fT3/fT4 and TFQI were independently associated with mCHS-defined frailty (odds ratio [OR] = 0.56 (per 0.1unit increase); 95% confidence interval [CI], 0.34–0.92 and OR = 2.68; 95%CI, 1.07–6.70, respectively). Using the receiver operating characteristic curve analysis, fT3/fT4 showed a modest, but relatively high area under the curve compared with TFQI, fT4, fT3, or thyroid-stimulating hormone, indicating a better discriminative ability.

**Conclusion:**

Indices of impaired peripheral and central sensitivity to thyroid hormones were independently associated with phenotype frailty based on the cardiovascular health study.

**Supplementary Information:**

The online version contains supplementary material available at 10.1186/s12877-025-06608-y.

## Background

Frailty is a state when a person becomes vulnerable to external stress due to aging [1]. Frailty is a strong risk factor for mortality and functional disability in the older population [2]. Another feature of frailty is its reversibility to the nonfrail state. Therefore, identifying a frail person in the early stages and intervene as early as possible after the diagnosis of frailty or at an earlier stage of prefrailty is essential. Various scales have been developed to diagnose frailty. The Cardiovascular Health Study (CHS) criteria developed by Fried et al. [1] included phenotype frailty. The Kihon Checklist (KCL) is based on the comprehensive geriatric assessment (CGA) [3].

The endocrine system maintains biological homeostasis through the regulation of several hormones. Recently, various endocrine system abnormalities have been associated with frailty. For instance, inappropriate secretion or sensitivity to glucocorticoid deficiency in growth hormones, sex steroids, and insulin are related to muscle weakness or frailty [4].

Some studies have reported an association between thyroid hormone dysfunction and frailty. In the normal state, thyroxine (T4) is deiodinized and converted into triiodothyronine (T3) in extrathyroidal tissue [5], the active form of the thyroid hormone. Most T4 and T3 proteins bind to various serum proteins, such as thyroxine-binding protein, transthyretin, and serum albumin, all of which have high binding affinity to T4 than T3 [6], which could account for the relatively shorter lives of T3 compared with T4.

Since protein-bound hormones have no bioavailability, measurement of free T3 (fT3) and free T4 (fT4), the non-protein-bound forms of the hormones, is necessary to evaluate the function of thyroid hormones. In patients with chronic inflammation or malnutrition, the conversion of T4 to T3 is suppressed. Instead, reverse T3 (rT3) is produced. rT3 has a low activity of the thyroid hormone on muscles, both of its synthetic and differentiative function and catabolic, degradative function [7].

A recent study has shown that subclinical hyperthyroidism (low thyroid-stimulating hormone (TSH) levels with normal fT4 levels) was associated with the prevalence of frailty according to the modified CHS criteria in older men [8]. A similar result was reported in a previous study, in which the frailty index was used to diagnose frailty [9]. In another report of Italian inpatient and outpatient older adults, lower fT3 levels were associated with higher risks of frailty, as defined by the modified CHS criteria [10].

The indices representing peripheral and central thyroid hormone sensitivity have recently been developed. However, impaired peripheral and central sensitivity to thyroid hormones could relate to frailty is unclear. Although a low fT3/fT4 ratio as an index of peripheral thyroid hormone sensitivity has been reported to be associated with a high prevalence of frailty, as assessed by the frailty index [11, 12], or multi-prognostic index in a cross-sectional study [13], few studies have evaluated the association between fT3/fT4 and phenotype frailty criteria developed by Fried et al. [1]. In contrast, a high Thyroid Feedback Quantile-based Index (TFQI), an index of impaired central sensitivity to thyroid hormones, and a low fT3/fT4 ratio are associated with cognitive impairment [14]. Moreover, no studies have examined the relationship between the TFQI and frailty, and it remains unknown which index reflecting thyroid hormone sensitivity is the best predictor of frailty.

At our institute, we established an outpatient clinic where we evaluated the frailty status of patients with cardiometabolic disease, such as diabetes mellitus and hypertension, which could be risk factors for cardiovascular disease and frailty [15]. At this clinic, we have reported various risk factors associated with frailty, such as growth/differentiation factor-15 (GDF-15) [16] as a serum marker and white matter abnormality on magnetic resonance imaging as an imaging marker [17] associated with the incidence of frailty.

In this cross-sectional study, we evaluated whether thyroid hormone sensitivity and frailty are independently associated in patients with cardiometabolic disease.

## Methods

### Participants

**Fig. 1:**
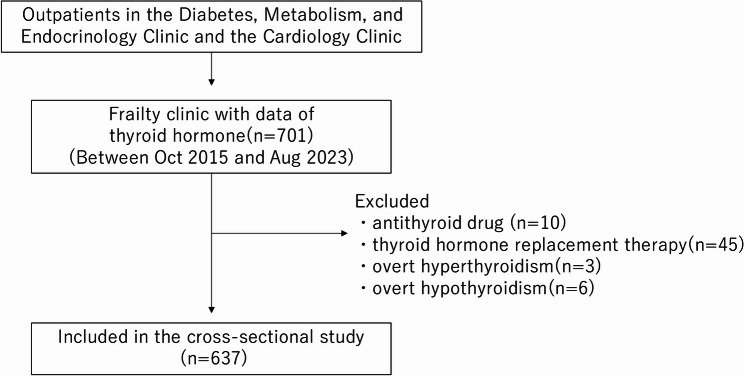
The flowchart of the participant selection process

A flowchart of the patient selection process is shown in Fig. 1. Older patients with cardiometabolic disease, such as diabetes and hypertension, suspected to be frail, participated and registered in the cohort study [15]. Seven hundred-one patients who underwent simultaneous evaluation of thyroid function and frailty status were enrolled. Of these, three patients who showed overt hyperthyroidism (TSH < 0.5µIU/ml, fT4 >21.9 pmol/L), six patients with overt hypothyroidism (TSH >5.0µIU/ml, fT4 < 11.6 pmol/L) [18], and 55 patients who were taking antithyroid drugs or thyroid hormone replacement therapy were excluded. Ultimately, data of 637 patients were included in the final analysis.

### Thyroid hormone and thyroid hormone sensitivity

The levels of fT4, fT3, and TSH were measured using a chemiluminescent immunoassay method, and fT3/fT4 was calculated as an index of impaired peripheral sensitivity to thyroid hormones in this study. The TFQI, an index of impaired central sensitivity to thyroid hormones, was calculated using the formula described by Laclaustra et al.’s report [19]. First, the mean and standard deviation were calculated for fT4 (µfT4, σfT4) and the natural logarithm of TSH (µLnTSH, σLnTSH) in these participants, where µfT4 = 15.3381, σfT4 = 2.63493, µTSH = 2.4341, σTSH = 1.72008, respectively. Next, the cumulative distribution function (CDF) of fT4 (cdfT4) and LnTSH (cdfLnTSH) was calculated as Φ(fT4-µfT4)/σfT4, and Φ(LnTSH-µLnTSH)/σLnTSH. TFQI was calculated using the formula cdfT4-(1-cdfLnTSH). TFQI ranges from − 1 to 1.

### Assessment of frailty

We adopted two indices for frailty diagnosis: the modified CHS (mCHS) criteria [15] and KCL [3]. Frailty diagnosed by each set of criteria has different clinical significance; CHS criteria were based on a phenotypic model, mainly evaluated physical frailty, whereas the KCL criteria were based on the CGA and could assess multidimensional aspects of frailty not only physical but also cognitive and social. We investigated which frailty criteria thyroid hormone sensitivity could influence the most. In the mCHS criteria, five items – unintentional weight loss, fatigue, low grip strength, low walking speed, and low physical activity – were evaluated, and those who tested positive for three or more items were identified as trials. Regarding the mCHS criteria, questions about physical activity were slightly modified from Fried’s original version as previously described [17]. The KCL consists of multiple domains, including functional disability, oral dysfunction, malnutrition, cognitive impairment, withdrawal, and depression. Those who tested positive for eight or more items were identified as frail.

### Other clinical evaluation

Cognitive function was evaluated using the Mini-Mental State Examination (MMSE), nutritional status using the Mini Nutritional Assessment Short-Form (MNA-SF), and depressive mood using the Geriatric Depression Scale 15 (GDS15) as previously described [15]. The International Physical Activity Questionnaire calculates physical activity (metabolic equivalents *min/week) [20].

### Ethics

Written informed consent was obtained from all participants. The study protocol was approved by the Ethics Committee of the Tokyo Metropolitan Institute for Geriatrics and Gerontology (approval no.: R15-20).

### Statistical analysis

Normality was assessed by Shapiro–Wilk’s test. Since most variables were found to be non-normally distributed, continuous variables were compared using the Mann-Whitney U test, whereas differences in the proportions of categorical variables were compared using the chi-squared test. The correlations between two continuous variables were evaluated using Spearman’s rank correlation coefficients. Multivariate logistic regression analysis was performed with frailty using mCHS or KCL criteria as the objective variables, setting fT4, fT3, TSH, fT3/fT4, or TFQI as explanatory variables, adjusting for age and sex (Model 1), MMSE score, GDS15 score, MNA-SF score, and physical activity (Mets*min/week) (Model 2), and further adding an inflammation-related marker, high-sensitivity C-reactive protein (hsCRP), to Model 3. In logistic regression analyses, cases with missing values were excluded using listwise deletion, while correlation analyses were conducted using pairwise deletion. Multicollinearity was tested by calculating variance inflation factor (VIF). Sensitivity analyses were performed by replacing MNA-SF with body mass index (BMI). Subgroup analyses were performed by stratifying participants according to the following variables: age (≥ 75 vs. <75 years), sex, and BMI (≥ 20 vs. <20). Interaction terms between thyroid hormone sensitivity and each of these variables were included to assess effect modification.

Next, we drew Receiver Operating Characteristic (ROC) curves to assess the discriminative ability of fT4, fT3, and TSH and the indices of impaired sensitivity to thyroid hormones. We determined the area under the curve and cut-off values using the Youden index method.

All statistical analyses were performed using SPSS version 26 (IBM Corp., Armonk, NY, USA). P values were considered statistically significant at *P* <.05.

## Results

### Clinical characteristics of patients

The clinical characteristics of the 637 patients whose data were used for the analysis are presented in Table 1. The mean age was 79 years, 64% were women, and the prevalence rates of diabetes, hypertension, and dyslipidemia were 43, 78, and 60%, respectively. The average fT3, fT4, and TSH were 4.0 pmol/L, 15.3 pmol/L, and 2.0µIU/ml, respectively.

**Table 1.**
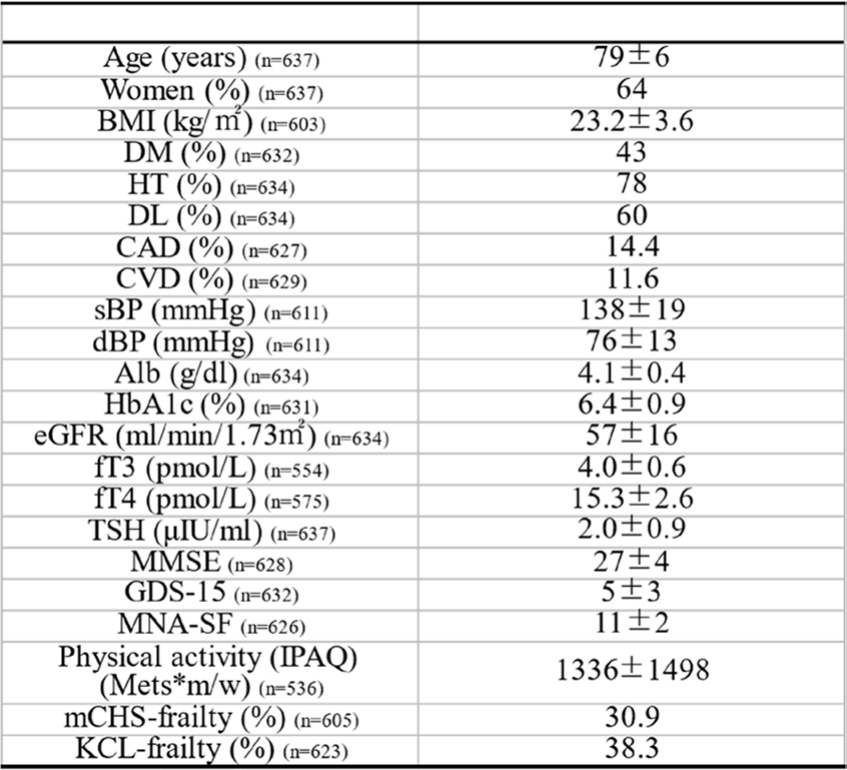
The clinical characteristics of the patients

Data are presented as numbers, means ± SDs or percentages. Abbreviations: *BMI* body mass index, *DM* diabetes mellitus, *HT* hypertension, *DL* dyslipidemia, *CAD* coronary artery discase, *CVD* cerebrovascular disease, *sBP* systolic blood pressure, *dBP* diastolic blood pressure, *Alb* albumin, *HbAlc* hemoglobin Alc, *eGFR* estimated Glomerular Filtration Rate, *fT3* free triiodothyronine, *fT4* free thyroxine, *TSH* thyroid-stimulating hormone, *MMSE* Mini-Mental State Examination, *GDS15* Geriatric Depression Scale 15, *MNA-SF* Mini Nutritional Assessment Short-Form, *IPAQ* International Physical Activity Questionnaire, *mCHS* modified Cardiovascular Health Study, *KCL* KihonChecklist- SD standard deviation

### Association between indices of thyroid hormone and its impaired sensitivity　and other clinical factors

Correlations between the indices of impaired sensitivity to thyroid hormones and the clinical parameters are summarized in Table 2**;** fT3/fT4 negatively correlated with age and GDS15 score and positively correlated with BMI, albumin, estimated glomerular filtration rate (eGFR), hsCRP, MMSE, MNA-SF scores, and physical activity. The TFQI positively correlated with glycated hemoglobin (HbA1c) and negatively correlated with albumin and eGFR.

**Table 2.**
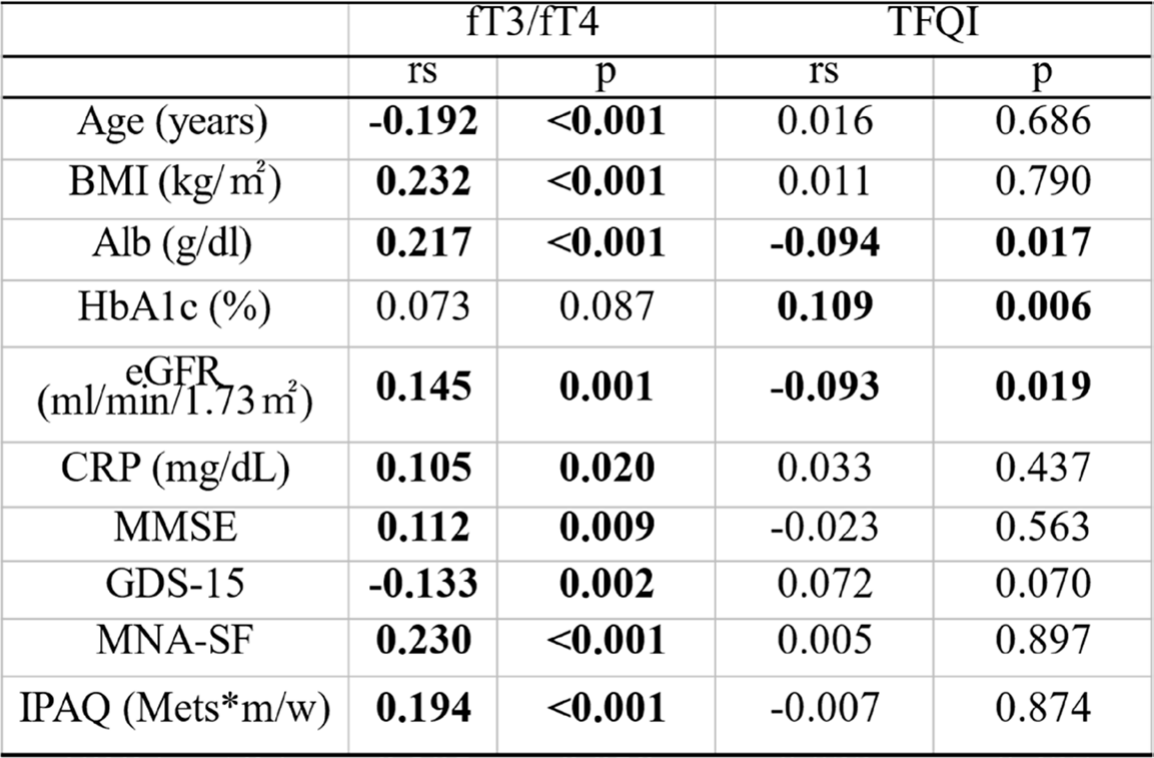
Correlations between the indices of impaired sensitivity to thyroid hormones and the clinical parameters

Abbreviations: *fT3* free triiodothyronine, *fT4* free thyroxine, *TFQI* Thyroid Feedback Quantile-based Index, *BMI* body mass index, *Alb* albumin, *HbA1c* hemoglobin A1c, *eGFR* estimated Glomerular Filtration Rate, *CRP* C-Reactive Protein, *MMSE* Mini-Mental State Examination, *GDS15* Geriatric Depression Scale 15, *MNA-SF* Mini Nutritional Assessment Short-Form, *IPAQ* International Physical Activity Questionnaire

### Indices thyroid hormone and its impaired sensitivity between mCHS- and KCL-defined frailty status

**Fig. 2:**
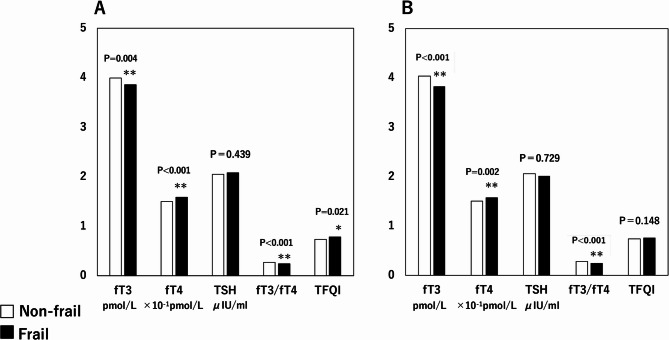
Comparisons of indices of thyroid hormone and its impaired sensitivity between frail and non-frail patients defined by (**A**) mCHS and (**B**) KCL criteria. Abbreviations: *fT3* free triiodothyronine, *fT4* free thyroxine, *TSH* thyroid-stimulating hormone, *TFQI* Thyroid Feedback Quantile-based Index

Patients with mCHS-defined frailty had significantly lower fT3, and fT3/fT4 and significantly higher fT4, and TFQI scores than those without frailty (*P* =.004, *P* <.001, *P* <.001, and *P* =.021, respectively. Figure 2A). Similarly, fT3 and fT3/fT4 were significantly lower, and fT4 was significantly higher in those with KCL-defined frailty than those without frailty (*P* <.001, *P* <.001, and *P* =.002, respectively Fig. 2B).

### Multivariate logistic regression analyses for the prevalence of frailty

A multivariate logistic regression analysis with frailty as the objective variable was performed (Table 3). When adjusted for age and sex (Model 1), the fT3/fT4 and TFQI scores were significantly associated with mCHS-defined frailty. After further adjustment for MMSE, GDS15, MNA-SF score, and physical activity (Model 2), the association of fT3/fT4 and TFQI with mCHS-defined frailty remained significant (fT3/fT4: odds ratio (OR) = 0.55 (per 0.1U increase), 95% CI: 0.34–0.87, *P* =.010 and TFQI: OR = 2.45, 95% CI: 1.05–5.75, *P* =.039). fT3/fT4, but not TFQI, was also independently associated with KCL-defined frailty (OR = 0.55, 95% CI: 0.34–0.88, *P* =.012). After further adjustment for hsCRP (Model 3), significant associations between fT3/fT4, TFQI, and mCHS-defined frailty persisted (*P* =.022 and *P* =.035, respectively). fT3/fT4, but not TFQI, was also independently associated with KCL-defined frailty (OR = 0.60, 95% CI: 0.36–1.00, *P* =.048). Using the sensitivity analyses, replacing MNA-SF with BMI did not affect the logistic regression results; however, the association between TFQI and mCHS-defined frailty became slightly less significant (Supplementary Table 1). Using subgroup analyses by age and BMI did not affect the results without significant interactions with these variables, while the sex difference found in the association between fT3/fT4 and mCHS-defined frailty (Supplementary Fig. 1). All VIFs or each covariate ranged between 1 and 2, indicating little concern for multicollinearity.

**Table 3.**
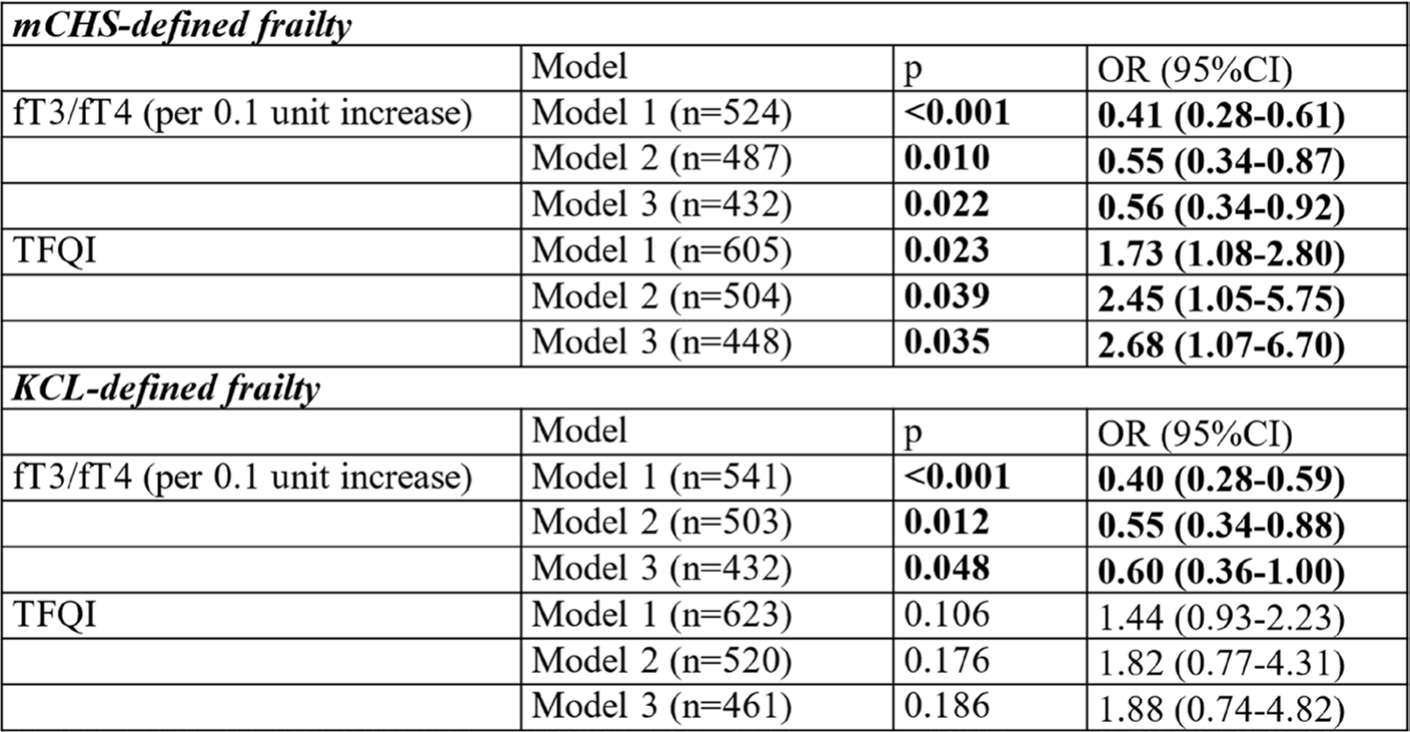
Multivariate logistic regression analyses for the prevalence of frailty

Model 1: Adjusted for age, sex. Model 2: Adjusted for age, sex, MMSE, GDS15, MNA-SF and physical activity Model 3: Adjusted for age, sex, MMSE, GDS15, MNA-SF, physical activity, and CRP Abbreviations: *mCHS* modified Cardiovascular Health Study, *KCL* kihon Checklist, *OR* odds ratio, *TFQI* Thyroid Feedback Quantile-based Index, *fT3* free triiodothyronine, *fT4* free thyroxine, *MMSE* Mini-Mental State Examination, *GDS15* Geriatric Depression Scale 15, *MNA-SF* Mini Nutritional Assessment Short-Form, *CRP* C-Reactive Protein

### ROC curves for prediction of mCHS- and KCL-defined frailty

**Fig. 3:**
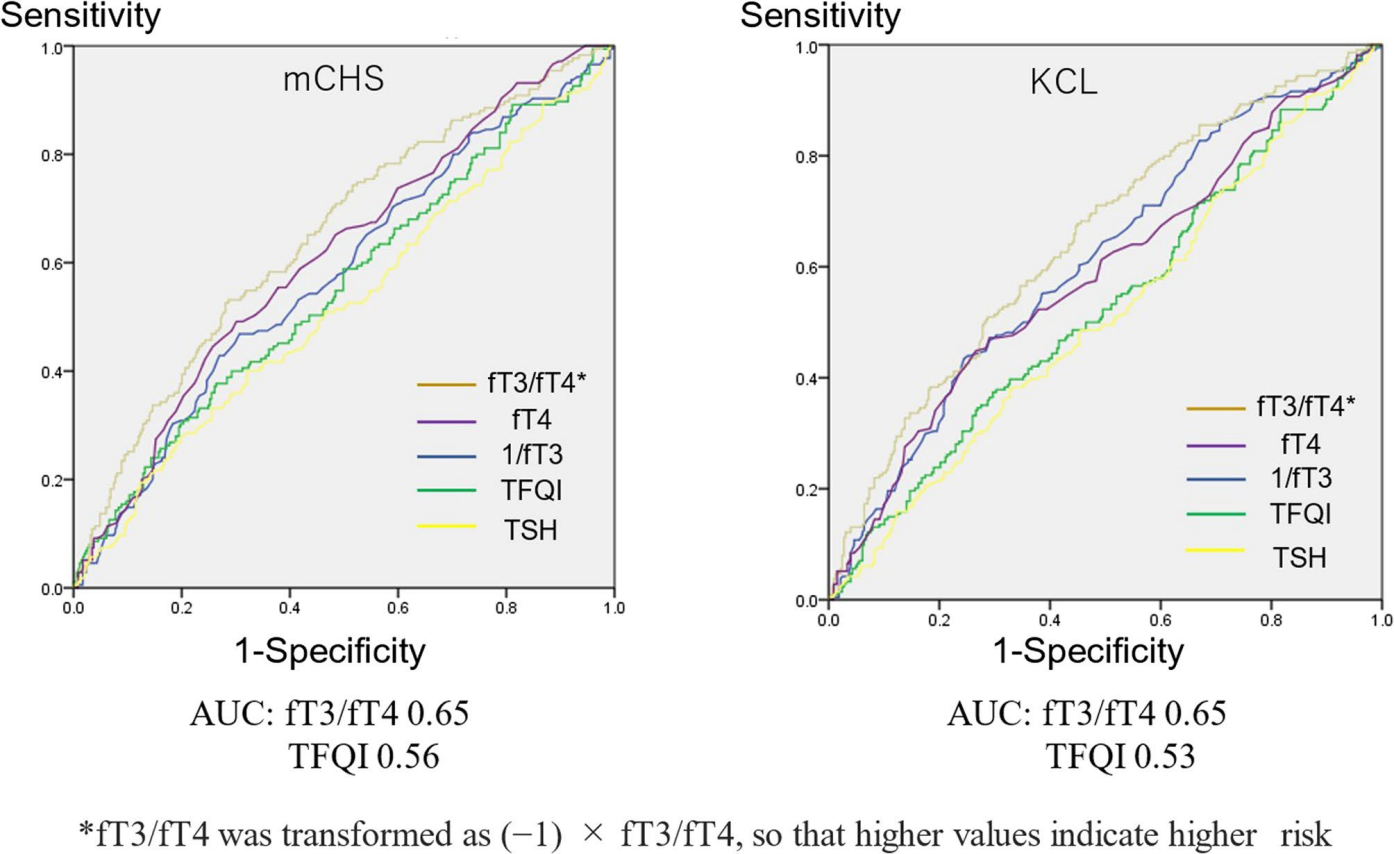
ROC curves for prediction of mCHS- and KCL-defined frailty. Abbreviations: *fT3* free triiodothyronine, *fT4* free thyroxine, *TFQI* Thyroid Feedback Quantile-based Index, *TSH* thyroid-stimulating hormone, *mCHS* modified Cardiovascular Health Study, *KCL* Kihon Checklist

The ROC curves are shown in Fig. 3. Considering the discrimination of mCHS-defined frailty, the area under the curve (AUC) values with fT3/fT4 were the highest (0.65), followed by fT4 (0.61), 1/fT3 (0.58), TFQI (0.56), and TSH (0.52). Similarly, the AUC values for discriminating KCL-defined frailty were the highest for fT3/fT4 (0.65), followed by 1/fT3 (0.61), whereas those for TFQI (0.53), fT4 (0.59), and TSH (0.51) were all lower.

## Discussion

In this study, we found that both markers for impaired peripheral and central sensitivity to thyroid hormones, fT3/fT4 and TFQI, were independently associated with mCHS-defined frailty after adjusting for covariates. Furthermore, we found that fT3/fT4 was the most useful marker for discriminating between both frailty types using ROC analyses.

fT3/fT4 is a marker of peripheral thyroid hormone sensitivity. T4 is converted into T3 in the peripheral tissue, and T3 has a higher thyroid hormone activity than does T4. In critical disease, converting T4 into T3, mediated by type 2 thyroxin deiodinase, is suppressed to avoid an over-catabolic state induced by T3. Impairment in thyroxin deiodinase activity is not merely a marker of frailty; however, it could also contribute to muscle loss. Dentice et al., reported using type 2 deiodinase knockout mice that it is associated with myogenesis and muscle regeneration [21]. Also, under these circumstances, the conversion of T4 into reverse T3, which has little thyroid hormone activity, is accelerated [22].

Our results on the association between fT3/fT4 and frailty are consistent with those in previous studies in which other frailty indices [11–13] or activities of daily living (ADL) [23] were evaluated. These studies have already shown impaired peripheral T4 deiodination correlates with frailty or ADL decline. However, the study settings and assessment methods for frailty in these previous studies differed from those used in our study. Two studies [11, 13] included mainly inpatients or residents of nursing care homes, whereas ours were outpatients. The participants in the other studies [12, 23] and home-residents in Okoye et al.’s study [11] were centenarians and/or semi-supercentenarians, whose ages were extremely higher than our participants. In the previous studies, frailty was assessed using the frailty index based on the deficit accumulation model or the Multidimensional Prognostic Index, a frailty tool according to the CGA. In this study, we used both the mCHS-defined frailty, a phenotype frailty, and the KCL-defined frailty based on the CGA. Our results suggest that the conversion of T4 to T3 could be suppressed in older outpatients with cardiometabolic disease, even those who were free from critical illness and that this suppression was associated with the phenotype frailty as well as the frailty based on the CGA.

The TFQI was first developed by Laclaustra et al. and reflects impaired central sensitivity to fT4 [19]. This impaired central sensitivity to fT4 indicates a TSH discrepancy from the estimated TSH level, which usually corresponds to the fT4 value. TFQI ranges between − 1 and 1, where 0 means TSH responds typically to fT4 value (normal sensitivity), and a plus value means TSH is higher than estimated (resistant to fT4). In contrast, a minus value means TSH is lower than estimated (sensitive to fT4). Recently, Yu et al. have reported that high TFQI, as well as low fT3/fT4, is associated with mild cognitive impairment in Chinese patients with type 2 diabetes [14]. In a Spanish cohort study, Alonso et al. reported that the group with the highest TFQI showed higher mortality [24].

No previous study has shown a positive association between TFQI and frailty, and this is the first report showing the association between TFQI and mCHS-defined frailty. In the cohort study mentioned above, Ostan et al. reported that both fT4 and TSH levels increased with age, indicating that TFQI would also increase [23].

These results suggest that impaired responsiveness to thyroid hormones is associated with frailty. This impairment could be induced not only by aging but also by comorbidity, inflammation, malnutrition and so on. Moreover, such an association between impaired responsiveness to hormones and frailty can be observed in other hormones, including resistance to insulin and cortisol suppression are associated with frailty [25, 26].

Various mechanisms are thought to be involved in the high prevalence of frailty among patients with low thyroid hormone sensitivity. First, they are associated with metabolic abnormalities. For instance, Lv et al. reported that the TFQI and a low fT3/fT4 ratio are associated with the prevalence of metabolic syndrome in the Chinese euthyroid population [27]. Moreover, some studies have shown that the sensitivity to thyroid hormones is associated with musculoskeletal abnormalities. Liu et al. showed that fT3/fT4 was positively and TFQI were negatively associated with bone mineral density [28] and Chen et al. showed that these indices were associated with the prevalence of osteoarthritis [29]. It is suspected to be associated with muscular dysfunction, which may be involved in the pathophysiology of frailty.

In this study, fT3/fT4 was associated with age, albumin, eGFR, MMSE, GDS15, MNA-SF, and physical activity, which also influence the incidence of frailty. As mentioned above, fT3/fT4 was reported to be negatively associated with cognitive function, which was reproducible in our study [14]. Under critical conditions, including malnutrition, the conversion from fT4 to rT3 increases, decreasing the fT3/fT4 ratio. Moreover, it is known that conversion to fT3 is associated with depressive mood. Wronski et al. reported that the fT3/fT4 ratio was lower in patients with anorexia nervosa and that among these patients, the fT3/fT4 ratio was further associated with the severity of depressive symptoms [30]. We could not find previous reports showing an association between fT4/fT3 and physical activity. However, the negative association between fT4/fT3 and physical activity in our study could partially be explained by reduced activity in those with cognitive impairment, malnutrition, or depressed mood.

The association between fT3/fT4 and KCL-defined frailty could also be attributed to the correlation between fT3/fT4 and most major KCL domains. However, the fact that the association between fT3/fT4 and KCL-defined frailty persists after adjusting for covariates such as MMSE and GDS15 indicates that factors other than these covariates may be involved in the association between fT3/fT4 and frailty.

The better discriminative ability of fT3/fT4 for frailty than TFQI could be because fT3/fT4 includes factors related to T3, which reflects the patient’s exhausted state more sensitively than TFQI, which is calculated without fT3. However, a possible explanation for the finding that TFQI was associated only with mCHS but not with the KCL is that the weight of “fatigue,” which could easily be influenced by thyroid dysfunction, was higher in mCHS (1/5) than in KCL (1/25).

In a meta-analysis of cross-sectional studies, inflammatory markers, such as higher levels of CRP, were associated with prefrailty and frailty. However, the relationship between thyroid function and inflammation is also bidirectional. In inflammatory disease, the conversion of T4 to T3 is suppressed. In contrast, when intracellular concentrations of T3 versus T4 in immune cells decrease, the production of reactive oxygen species and cytokine release increases in monocytes and macrophages [31]. However, the association between the indices of impaired sensitivity to thyroid hormones and mCHS-defined frailty persists after adjustment for CRP in multivariate analysis, suggesting that the effect of impaired sensitivity to thyroid hormones on mCHS frailty is not entirely mediated by inflammation. The association between fT3/fT4 and KCL-defined frailty also persisted after adjustment of CRP. However, as it has been shown that systemic inflammation is associated with various items in CGA, such as cognitive impairment, depressive mood [32], and malnutrition [33], the association may have been slightly attenuated (Table 3).

In the subgroup analyses by age or BMI, no significant interactions were found in relation to thyroid hormone sensitivity and frailty. However, the association between fT3/fT4 and mCHS-defined frailty was significantly stronger in male participants, whereas its link with KCL-defined frailty was evident in females. The reason for this discrepancy is unclear. Jiang et al. reported that the association between thyroid hormone sensitivity and cardiovascular–kidney–metabolic syndrome was stronger in male patients, suggesting the greater susceptibility of men to metabolic derangement compared with women [34]. Our findings may reflect sex-specific effects of thyroid hormones on peripheral organs. In men, who generally have greater muscle mass, impaired thyroid hormone action may predominantly affect muscle synthesis and differentiation, whereas in women, these muscle-related effects may be less pronounced, and the effect on other organs such as the brain might play a larger role in the development of KCL-defined frailty.

The strength of this study is that we evaluated the association between impaired sensitivity to peripheral and central thyroid hormones and the two types of frailty defined by different concepts in a large number of outpatients. We examined the association between two types of frailty and fT3/fT4, and TFQI simultaneously, which provided additional insights into the complex interplay between thyroid function and aging-related vulnerability.

fT3/fT4 is a simple marker calculated using only values obtained from a single blood sample. We found this simple, valuable marker for predicting the prevalence of frailty in patients with cardiometabolic disease with a higher potential to progress to frailty. Notably, indices of impaired sensitivity to thyroid hormones could pose additional risks even in populations susceptible to frailty. Whether this additional influence is similar in the general older population needs to be clarified.

However, this study has some limitations. First, because this was a cross-sectional study, the causal relationship between impaired sensitivity to thyroid hormones and frailty remains unclear. As this study employed a cross-sectional design to simply examine whether thyroid hormone sensitivity and frailty are independently associated, we did not conduct a mediation analysis. However, to explore potential mediating pathways, we are planning a longitudinal study with a larger sample size in which mediation analysis will be conducted to further elucidate the causal relationships. Second, we selected only fT3/fT4 as index for peripheral thyroid hormone sensitivity. Although it reflects a state where peripheral tissues are resistant to thyroid hormone, it may also decrease in a state where activity of type 2 and 3 deiodinase modified for other reasons. Other indices for peripheral thyroid hormone sensitivity, such as rT3, should have been simultaneously evaluated to confirm the impairment. Third, the TFQI should be ideally calculated, assuming that fT4 and TSH levels are normally distributed. However, it was not distributed normally, as we excluded overt hyperthyroid and hypothyroid patients, which could have affected the reliability and low prediction capacity of the TFQI for frailty. Fourth, as we showed above, even the AUC of ROC curve with fT3/fT4, which most significantly predicted frailty was still as low as 0.65. Thus, diagnosing frailty only by this information may be a risk and it could be recommendable in clinical practice to combine these with other various data individually. Lastly, this study was performed at a single institution, and the influence of race and ethnicity remains unclear. Particularly, as the validation of KCL had been done only in a limited number of countries other than Japan, such as China [35] and Thailand [36], it is unclear whether the result of KCL in our study can be directly generalized to other countries or ethnicities. Moreover, as described above, this study included a limited number of patients with cardiometabolic disease. A multicenter, worldwide study of community-dwelling older adults should be conducted to generalize our results.

In conclusion, we found that two markers of impaired sensitivity to thyroid hormones, fT3/fT4 and TFQI, were associated with the prevalence of phenotype frailty in older patients with cardiometabolic disease. Among these, fT3/fT4 was the most appropriate markers for predicting frailty. Furthermore, longitudinal studies are needed to clarify whether these markers could indicate the incidence of frailty and whether frailty prevention interventions, such as exercise, could modify the markers of impaired sensitivity to thyroid hormones.

## Supplementary Information

Supplementary Material 1

Supplementary Material 2

## Data Availability

The datasets used and/or analysed during the current study are available from the corresponding author on reasonable request.

## Abbreviations

AUC: Area under the curve
BMI: Body mass index
CDF: Cumulative distribution function
CGA: Comprehensive geriatric assessment
eGFR: Estimated Glomerular Filtration Rate
fT3: Free triiodothyronine
fT4: Free thyroxine
GDF-15: Growth differentiation factor- 15
GDS15: Geriatric Depression Scale 15
HbA1c: Glycated hemoglobin
hsCRP: High sensitivity C -reactive protein
KCL: Kihon Checklist
mCHS: Modified cardiovascular health study
MMSE: Mini-mental state examination
MNA-SF: Mini nutritional assessment short-form
ROC: Receiver operating characteristic
rT3: Reverse T3
T3: Triiodothyronine
T4: Thyroxine
TFQI: Thyroid feedback quantile-based index
TSH: Thyroid-stimulating hormone
