# Supplementary material for: Impaired sensitivity to thyroid hormones is associated with frailty in older patients with cardiometabolic disease
Source: BMC Geriatr. 2025 Nov 25;26:6. doi: 10.1186/s12877-025-06608-y (PMC12763869; doi:10.1186/s12877-025-06608-y)
Supplement: Supplementary file 1 — Supplementary Material 1 [file 12877_2025_6608_MOESM1_ESM.pdf]

Supplementary Table1：**Multivariate logistic regression analyses for the prevalence of frailty replacing MNA-SF with BMI**

mCHS-defined frailty			
	Model	p	OR (95%CI)
fT3/fT4 (per 0.1unit increase)	Model 1 (n=524)	**<0.001**	**0.41 (0.28-0.61)**
	Model 2 (n=488)	**0.002**	**0.48 (0.30-0.76)**
	Model 3 (n=433)	**0.003**	**0.46 (0.28-0.77)**
TFQI	Model 1 (n=605)	**0.023**	**1.73 (1.08-2.80)**
	Model 2 (n=505)	0.090	2.05 (0.90-4.68)
	Model 3 (n=446)	0.071	2.29 (0.93-5.60)
KCL-defined frailty			
	Model	p	OR (95%CI)
fT3/fT4 (per 0.1unit increase)	Model 1 (n=541)	**<0.001**	**0.40 (0.28-0.59)**
	Model 2 (n=504)	**0.004**	**0.50 (0.31-0.80)**
	Model 3 (n=449)	**0.015**	**0.53 (0.32-0.89)**
TFQI	Model 1 (n=623)	0.106	1.44 (0.93-2.23)
	Model 2 (n=521)	0.262	1.63 (0.70-3.80)
	Model 3 (n=462)	0.269	1.68 (0.67-4.22)

Model 1: Adjusted for age, sex

Model 2: Adjusted for age, sex, MMSE, GDS15, BMI and physical activity

Model 3: Adjusted for age, sex, MMSE, GDS15, BMI, physical activity, and CRP

Abbreviations: mCHS, modified Cardiovascular Health Study; KCL, kihon Checklist; OR, odds ratio; TFQI, Thyroid Feedback Quantile-based Index; fT3, free triiodothyronine; fT4, free thyroxine; MMSE, Mini-Mental State Examination; GDS-15, Geriatric Depression Scale 15; BMI, body mass index; CRP, C-Reactive Protein
