# Supplementary material for: Impaired sensitivity to thyroid hormones is associated with frailty in older patients with cardiometabolic disease
Source: BMC Geriatr. 2025 Nov 25;26:6. doi: 10.1186/s12877-025-06608-y (PMC12763869; doi:10.1186/s12877-025-06608-y)
Supplement: Supplementary file 2 — Supplementary Material 2 [file 12877_2025_6608_MOESM2_ESM.pdf]

Supplementary Figure 1. Multiple logistic regression analyses stratified by age, sex, and BMI (Model 2)

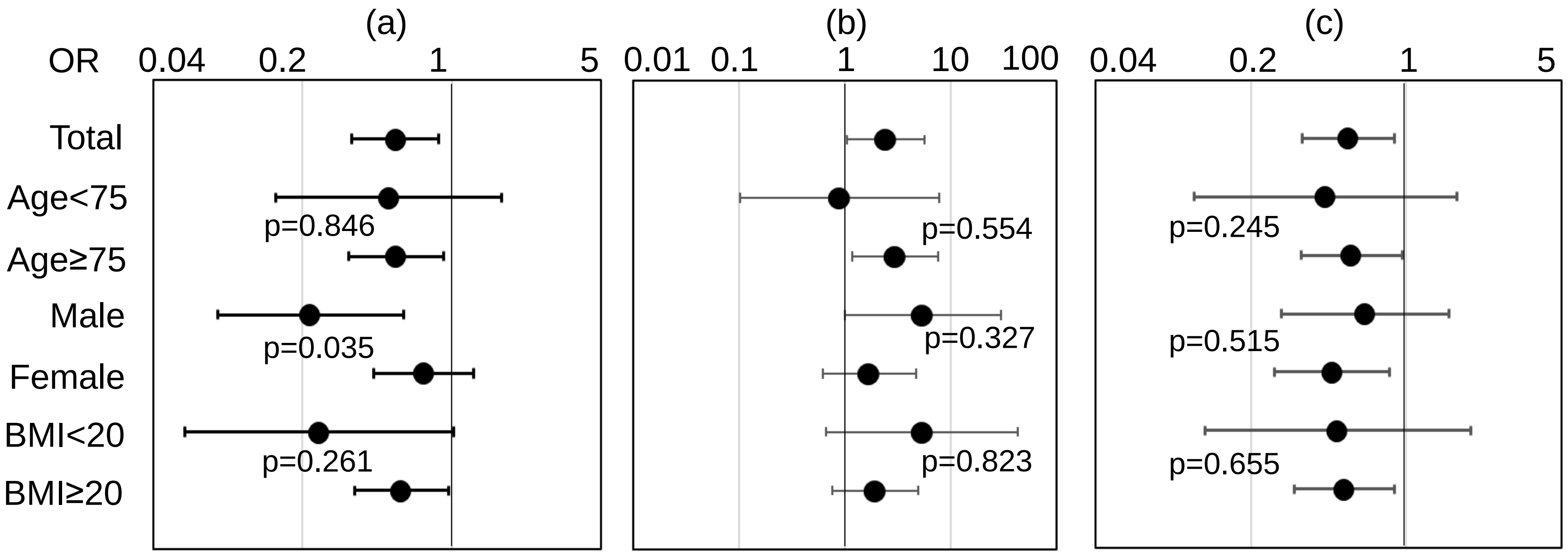

Associations between (a) fT3/fT4 and mCHS-defined frailty, (b) TFQI and mCHS-defined frailty, and (c) fT3/fT4 and KCL-defined frailty are shown. p-values for interaction are presented.
